# Drylands soil bacterial community is affected by land use change and different irrigation practices in the Mezquital Valley, Mexico

**DOI:** 10.1038/s41598-018-19743-x

**Published:** 2018-01-23

**Authors:** Kathia Lüneberg, Dominik Schneider, Christina Siebe, Rolf Daniel

**Affiliations:** 1 Departamento de Edafología, Instituto de Geología, Universidad Nacional Autónoma de México, Ciudad Universitaria, Mexico City, Mexico; 2 Genomic and Applied Microbiology and Göttingen Genomics Laboratory, Institute of Microbiology and Genetics, Universität Göttingen, Göttingen, Germany

## Abstract

Dryland agriculture nourishes one third of global population, although crop irrigation is often mandatory. As freshwater sources are scarce, treated and untreated wastewater is increasingly used for irrigation. Here, we investigated how the transformation of semiarid shrubland into rainfed farming or irrigated agriculture with freshwater, dam-stored or untreated wastewater affects the total (DNA-based) and active (RNA-based) soil bacterial community composition, diversity, and functionality. To do this we collected soil samples during the dry and rainy seasons and isolated DNA and RNA. Soil moisture, sodium content and pH were the strongest drivers of the bacterial community composition. We found lineage-specific adaptations to drought and sodium content in specific land use systems. Predicted functionality profiles revealed gene abundances involved in nitrogen, carbon and phosphorous cycles differed among land use systems and season. Freshwater irrigated bacterial community is taxonomically and functionally susceptible to seasonal environmental changes, while wastewater irrigated ones are taxonomically susceptible but functionally resistant to them. Additionally, we identified potentially harmful human and phytopathogens. The analyses of 16 S rRNA genes, its transcripts and deduced functional profiles provided extensive understanding of the short-term and long-term responses of bacterial communities associated to land use, seasonality, and water quality used for irrigation in drylands.

## Introduction

Drylands are defined as regions with arid, semi-arid, and dry sub humid climate with an annual precipitation/evapotranspiration potential ratio (P/PET)^1^ ranging from 0.05 to 0.65^2^. This ecosystem covers 40% of the world’s land surface^3^ and its extension could increase as the global surface temperature rises and temporal as well as spatial patterns of precipitation are modified due to climate change^4,5^. The crop production in drylands is important for maintaining the food security regionally, supporting one third of the global population^3^. The climatic conditions in these regions limit soil moisture and constrain farming to the rainy season (1–179 days)^2^ if irrigation is not feasible. As world population grows, crop demand raises and fresh water resources become scarce. Thus, the only way to maintain agricultural productivity in these areas is by using treated or untreated wastewater for field irrigation^3,6–8^.

In recent years, the knowledge on soil microbial communities in drylands has increased as they participate in critical processes needed to maintain soil quality and ecosystem functioning. For instance, soil microbes are important for organic matter decomposition and depend on the quality and amount of the plant residues that are incorporated into the soil. They are also responsible for cycling essential elements, such as nitrogen (N), carbon (C), phosphorus (P) and sulphur (S), which are converted to their inorganic forms^9,10^. The structure and diversity of soil microbial communities is driven by the soil physicochemical characteristics, such as pH, soil moisture, organic C and nutrient content; which are influenced by environmental parameters, as climate, land use type, and management^11–14^.

The land use change with agricultural purposes in drylands, as in many other ecosystems, modifies the quantity and quality of organic matter, diminishing soil carbon stocks^15,16^. In addition, the use of wastewater for crop irrigation modifies soil physicochemical properties. Although wastewater irrigation particularly improves the availability of labile organic carbon and nutrients in soil (N and P)^6^, this practice is pernicious as it adds soluble salts, heavy metals, pharmaceuticals, and microbial pathogens to the soil^6,17–19^. The World Health Organization (WHO)^20^ recommends the treatment of wastewater used for irrigation to reduce the concentration of pathogens and chemicals or the restriction of irrigation with untreated or primary treated wastewater to non-food crops, food crops that are processed before consumption and food crops that have to be cooked. However, irrigation with untreated wastewater is still a common practice in many countries^21^. In fact, around 6–20 million ha around 3 out of 4 cities in the developing world use untreated wastewater for irrigation^22^.

Soil microbial communities affected by land use change and management have been widely studied in forest, jungle, and grassland ecosystems, frequently contrasting between tilled or fertilized systems^10,12,14^. Surveys targeting soil microbial communities of natural drylands^23^ and their shift to agroecosystems with different managements are rare^24^. Even less studies exist targeting the effect of the water quantity and quality used for crop irrigation on the soil bacterial community composition and functions in dry regions^19,25^.

Transformation of drylands into agricultural fields resulted in a radical shift of soil bacterial community composition^24,26^. In hyper arid desserts, agriculture conversion resulted in an increase of bacterial diversity^24^, while in semiarid regions in a decrease^26^. It has also been suggested that agricultural management and seasonal changes influence the bacterial communities in dryland soil^27,28^. Watering of crops has shown a beneficial effect on the soil microbial community compared to rainfed systems, enhancing soil microbial biomass and respiration^28,29^. The use of treated and untreated wastewater for crop irrigation in dryland areas has shown impact on the bacterial community composition, but not in bacterial diversity^19,25,30^. In dryland soil the primary determinants of bacterial community structure are soil moisture and pH, and to a lesser extent organic carbon content^23,24,31^.

The objective of this study was to determine the impact on soil bacterial community composition and diversity of converting natural semi-arid shrubland to farmland. We particularly were interested in studying the influence of water quantity and quality used for crop irrigation. The study was conducted in the Mezquital Valley, which is a semi-arid region (P/PET 0.32). It is located in central Mexico and considered to be the largest area in the world that irrigates crops with untreated wastewater^32,33^. The Mezquital Valley provides representative conditions to study soil bacterial communities under different land use systems in drylands including preserved natural vegetation (shrubland) in the upper piedmonts, rainfed agriculture or irrigated agriculture in the middle to lower piedmonts, and valley bottoms. Three different water qualities are used for irrigation: freshwater, untreated wastewater stored in a dam for at least 3 months and untreated wastewater. The soil of this area provides two important services, on the one hand crop production, in 2014 more than 4 million tons of fodder crops and vegetables were produced in this area^34^. On the other hand, filtering the untreated wastewater used for irrigation to recharge the aquifer. This aquifer is used by the surrounding population for consumption and recreational activities.

In this study, we used amplicon-based sequencing of 16S rRNA genes and transcripts to characterize the soil bacterial community composition and diversity from natural dryland vegetation (shrubland), fields under rainfed and irrigation agriculture, during the dry and rainy seasons. We employed DNA-based and RNA-based analysis to assess total and potentially active bacterial community, respectively. The presence of 16S rRNA transcripts is a proxy for active community members, as it indicates the potential of an (micro-)organism to synthesise proteins^35,36^. Additionally, we correlated the soil properties of each studied system with the changes in the structure of soil bacteria. We hypothesize that differences in soil physicochemical properties derived from changes in land use from natural dryland to agricultural land under different systems influence the soil bacterial composition, diversity and function. We hypothesize further that (a) bacterial composition is primarily driven by soil moisture, as, in semiarid climates, moisture is the primary limiting factor for bacterial growth and activity, (b) the communities from shrubland and rainfed soil are similar as both remain dry for several months, (c) communities from soil irrigated with wastewater (dam-stored or untreated) are more similar to each other compared to freshwater irrigated soils. In addition, we predict a higher bacterial diversity in shrubland soil than in agricultural systems, as this has previously been observed in semiarid regions under land use change. We also expected differences in the composition of the communities during dry and rainy season. Our study contributes to better understand the impact of land use transformation and different agricultural systems (water quality and quantity for irrigation) on total and active bacterial communities in semiarid ecosystems.

## Results and Discussion

### Land use change and water quality use for irrigation alter soil properties

**Figure 1:**
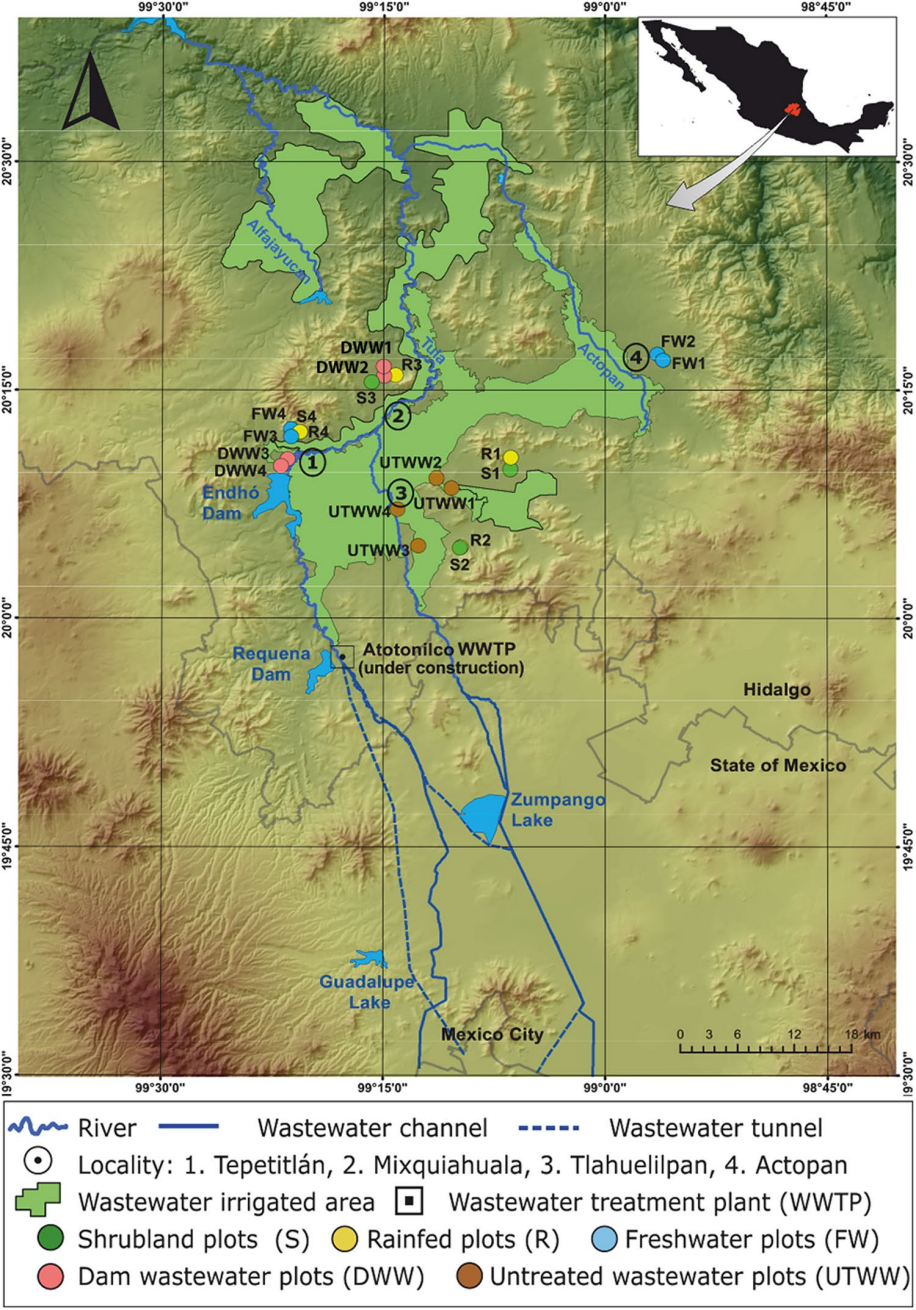
Location of the study sites within the Mezquital Valley (Mexico). Map was created with ESRI 2011. ArcGIS Desktop: Release 10. Redlands, CA: Environmental Systems Research Institute.

**Figure 2:**
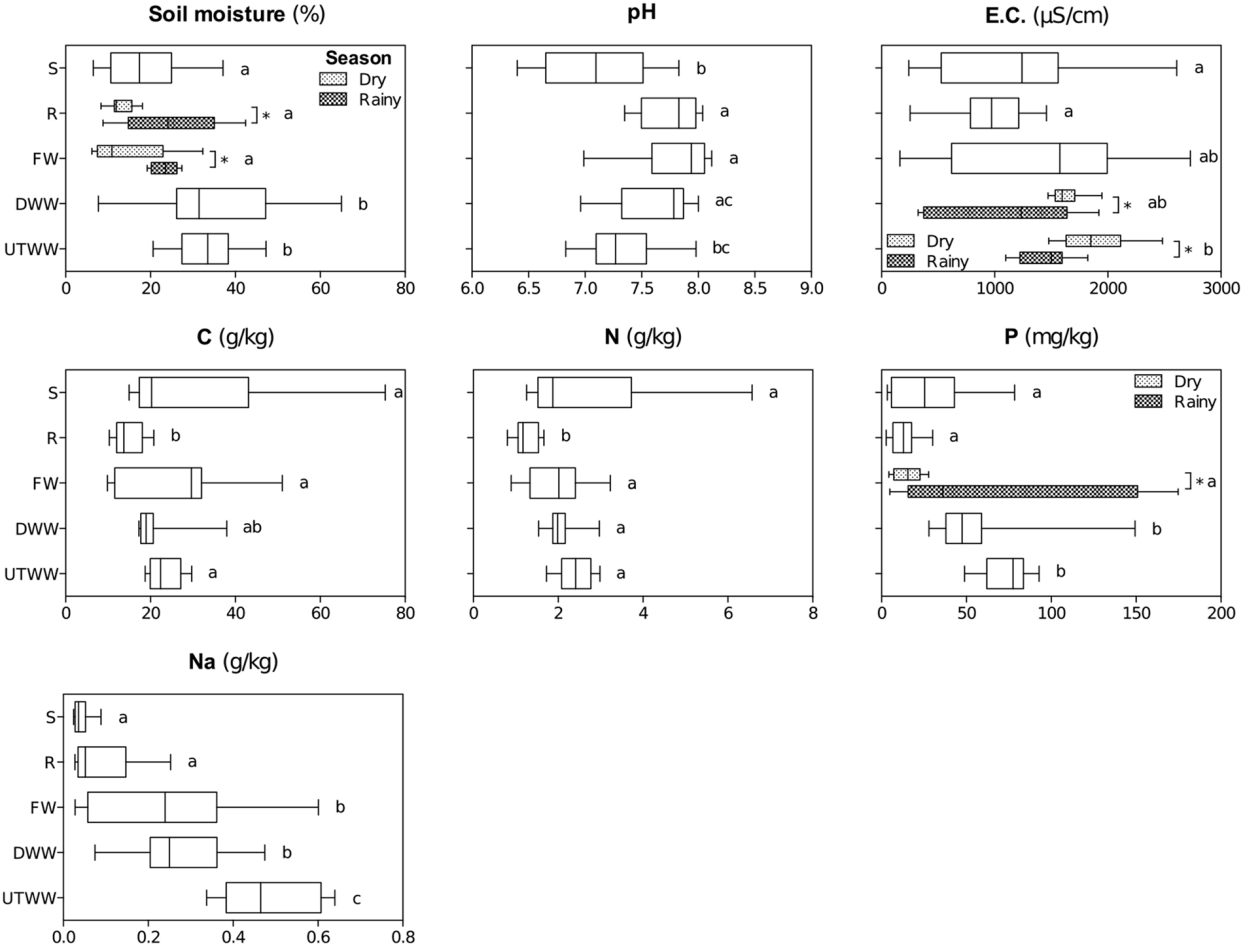
Soil properties in Shrubland (S), Rainfed (R), Freshwater (FW), Dam wastewater (DWW) and Untreated wastewater (UTWW) systems, during the dry and rainy season. Box are extended from the 25^th^ to 75^th^ percentiles, the line in the box is plotted at the median. Whiskers represent the smallest and the largest value. Kruskal-Wallis and Dunn’s tests were used to determined differences among land use systems, and permutation test for differences between seasons. Only parameters differing significantly between seasons in each land use system are shown, if they did not differ, samples of both seasons were merged.

We analyzed soil under five land use systems: xerophytic shrubland (S), agricultural fields under rainfed (R), fresh water irrigation (FW), dam-stored wastewater irrigation (DWW) and untreated wastewater irrigation (UTWW) (Fig. 1). The soils are classified as Phaeozems with high clay content (on average 44.7%). The soil properties differed significantly among land use systems and some properties such as moisture, electric conductivity and P content also between seasons (Fig. 2 and Supplementary material Fig. S1). Soil moisture was higher in soils irrigated with DWW and UTWW with more than 30%, while the rest of the land use systems had less than 19%. Rainfed and FW showed higher moisture during the rainy season. The pH ranged from 7.1 to 7.8, being the lowest in shrubland and the highest in rainfed and FW. The increase and decrease of soil pH due to irrigation with different types of wastewater has been reported earlier^8,37^. The increase is caused by additional input of exchangeable cations, mostly sodium^38^, and the decrease by large ammonium-nitrogen input. In the rainfed, soil N and C content was lowest (1.6 and 12 g kg^−1^, respectively) compared to the other systems (on average 2.1 and 25 g kg^−1^, respectively). The concentration of calcium in the ion exchange complex of the soil decreased in soil irrigated with untreated wastewater, as the high input of sodium displaces calcium from soil^6^. Several soil properties showed an increasing tendency from rainfed to UTWW, such as electrical conductivity, and the amount of sodium, potassium, magnesium and phosphorous. It has previously been reported that flood irrigation and the use of different types of wastewater increase soil salinity^8,39^. The electrical conductivity was significantly lower during the rainy season in the wastewater-irrigated soils (DWW and UTWW), which can be explained by dilution of wastewater. P was higher during the rainy season in FW, probably due to fertilization.

### General characteristics of the 16S rRNA datasets

To analyze the bacterial community structure and diversity, DNA and RNA were isolated from a total of 80 soil samples derived from all analyzed systems. We isolated DNA from all 80 samples and RNA from 52. Despite several attempts with modified conditions, the isolation of high quality RNA or cDNA synthesis in sufficient amount from four shrubland, 13 rainfed, two FW, eight DWW and one UTWW samples failed. After removal of low-quality sequences and singletons amplicon-based analysis of the V3-V4 region of the 16S rRNA resulted in 5,040,696 (DNA) and 3,370,420 (RNA) high-quality sequences. The number of sequences per sample ranged from 18,976 to 299,941 (DNA) and from 19,618 to 487,021 (RNA). After rarefaction analysis with the minimal amount of sequences per sample (18,900 DNA, 19,600 RNA) we obtained 12,854 OTUs from DNA (2,544 ± 238 per sample) and 12,182 from RNA (2,590 ± 337 per sample). The Good’s coverage index for DNA and RNA of 0.95 (±0.007) indicates that the datasets enclose all major bacterial groups inhabiting the studied land use systems. Rarefaction curves can be found in the Supplementary Material Fig. S2. Because 13 rainfed samples were lacking at RNA level it was not possible to draw conclusions between seasons of the active bacterial community for this land use system.

### Land use systems impact bacterial diversity

Bacterial diversity (Shannon index *H*’ and Faith’s phylogenetic diversity index PD) responded to land use change and water used for irrigation (p ≤ 0.05, Fig. S3). Total (DNA-based) and potentially active communities (RNA-based) did not show the same pattern. The diversity of the total bacterial community was higher in FW (*H*’ 9.67, PD 143) than the corresponding active communities (*H*’ 9.3, PD 135). The opposite was recorded for UTWW (total: *H*’ 9.4, PD 134; active: *H*’ 9.5, PD 145.5). In contrast to our hypothesis (c), the diversity of the natural dryland community was only higher compared to that of the rainfed system but not than those of the other systems. These results partially agree with previous studies that have reported higher diversity in agricultural soil than in natural drylands^24^. Additionally, wastewater irrigated soil has previously shown higher bacterial and enzymatic activity as a result of the nutrients and organic matter provided by wastewater^29^. A previous work studying Mezquital Valley soil^19^ reported no significant differences in the total bacterial richness and diversity between rainfed and UTWW, but a richer soil bacterial community during the dry season. Our results indicate that only FW exhibited a richer bacterial diversity during the dry season, which was related to the available P (r_s_ = −0.24, p ≤ 0.05). The differences between both studies might be due to a lower survey size and lower amount of analyzed samples in the other study.

### Impact of soil parameters on bacterial community composition and diversity

**Figure 3:**
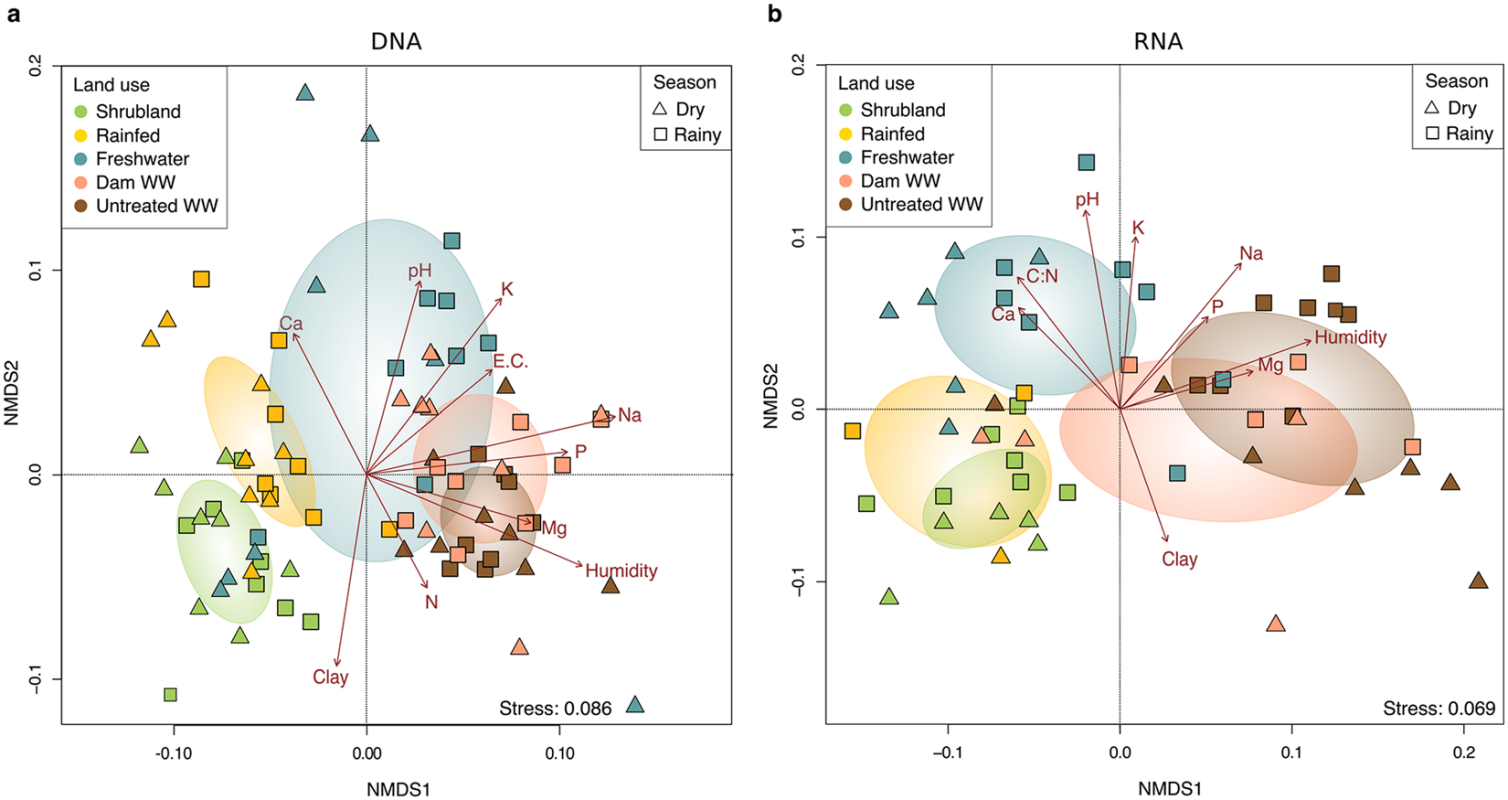
Non-metric multidimensional scaling (NMDS) of total (**a**) and potentially active (**b**) bacterial community composition of all samples from land use systems, shrubland, rainfed, freshwater, dam wastewater and untreated wastewater irrigation and dry and rainy season based on weighted Unifrac^87^ distance matrices. Environmental parameters that were significantly correlated (p ≤ 0.05) to bacterial community structure are indicated by arrows.

As indicated by ANOSIM test (p = 0.01), the composition of the total and active bacterial communities differed by land use system. The (dis)similarities are driven by conversion of shrubland to agricultural land use systems, especially from those under monthly irrigation. We could observe that the communities from shrubland and rainfed systems are similar, while the communities irrigated with wastewater (DWW and UTWW) are even more similar to each other, as they cluster nearby (Fig. 3). The FW community distributed between these two groups, representing an intermediate land use system, as we hypothesized. Despite RNA clustering was less condense, the general pattern remained the same for DNA and RNA. It is important to notice that the crop type or vegetation cover is the consequence of the land use system and the season. Shrubland soil presents the same vegetation cover trough out the year, while the agricultural systems are cropped with maize during the rainy season and during the dry season the plots were left fallow or cropped with oat, grass or alfalfa. Hence, after evaluating the structure of the community differentiating by season, we observed a stronger effect by the crop type/vegetation cover during the dry season than in the rainy season (Supplementary material Fig. S4). We observed differences between seasons (ANOSIM test, p = 0.01) in the structure of the total and potentially active community of the FW system and in the potentially active community of the UTWW system. These results were influenced by different soil properties, and in the case of the potentially active communities by the crop type present in the different seasons (Supplementary material Fig. S5). Cover crops have shown to modify the soil microbial community^28^.

We tested the correlation of soil characteristics and fitted them onto the ordination to determine what properties were correlated to the bacterial community compositions. Exchangeable sodium content exhibited the strongest impact on the total bacterial community (R^2^ = 0.504, p = 0.001), followed by soil moisture (R^2^ = 0.416, p = 0.001; Fig. 3a). The structure of the potentially active community was influenced by soil moisture and pH (R^2^ = 0.557, 0.553, respectively, p = 0.001), followed by sodium content (R^2^ = 0.48, p = 0.001; Fig. 3b). These results are consistent with previous reports of soil microbial communities in drylands^24,40^, in which water content was identified as the limiting factor for bacterial growth and activity^41^. In addition, water content changed drastically by introducing crop irrigation, excess soil water content results in limited O_2_ diffusion, which reduces microbial aerobic activity, but could increase the activity of anaerobes^42^. Soil pH has been widely reported to be the strongest driver of bacterial community structure^43–45^. In the soils of the Mezquital Valley this trend is preserved despite the small pH range (7.1 to 7.8). The pH is directly or indirectly related to many soil characteristics including soil moisture, salinity and nutrient availability^43^. Previous studies^42^ have shown that high concentrations of soil soluble salts, such as Na, affect microbial community structure, as they reduce the osmotic potential of microbial cells. Hence, they can adapt to low osmotic potentials by accumulating osmolyts, which is energetically costly^42^. Higher sodium content in soil irrigated with wastewater has been reported to have negative effects on basal respiration and adenylate energy charge (AEC), while the denitrification capacity increased^29^. These demonstrate the ability some bacterial taxa have to adapt to or tolerate stress cause by Na soil content.

Shannon index was positively correlated to soil pH at the entire community level (r_s_ = 0.34, p ≤ 0.05; Supplementary material Table S2). This effect of soil pH on bacterial diversity has been reported earlier in a multiple ecosystem type study^43^ and in forest and grasslands soil^45^; the latter study showed higher diversity under slight alkaline conditions, similar to what we observed. To our knowledge, the effect of soil pH has not been shown for irrigation systems before. Interestingly, the diversity of the active community was positively correlated to soil moisture and sodium content (r_s_ = 0.36 and 0.41, respectively; p ≤ 0.05; Supplementary material Table S2).

### General structure of the soil bacterial communities

**Figure 4:**
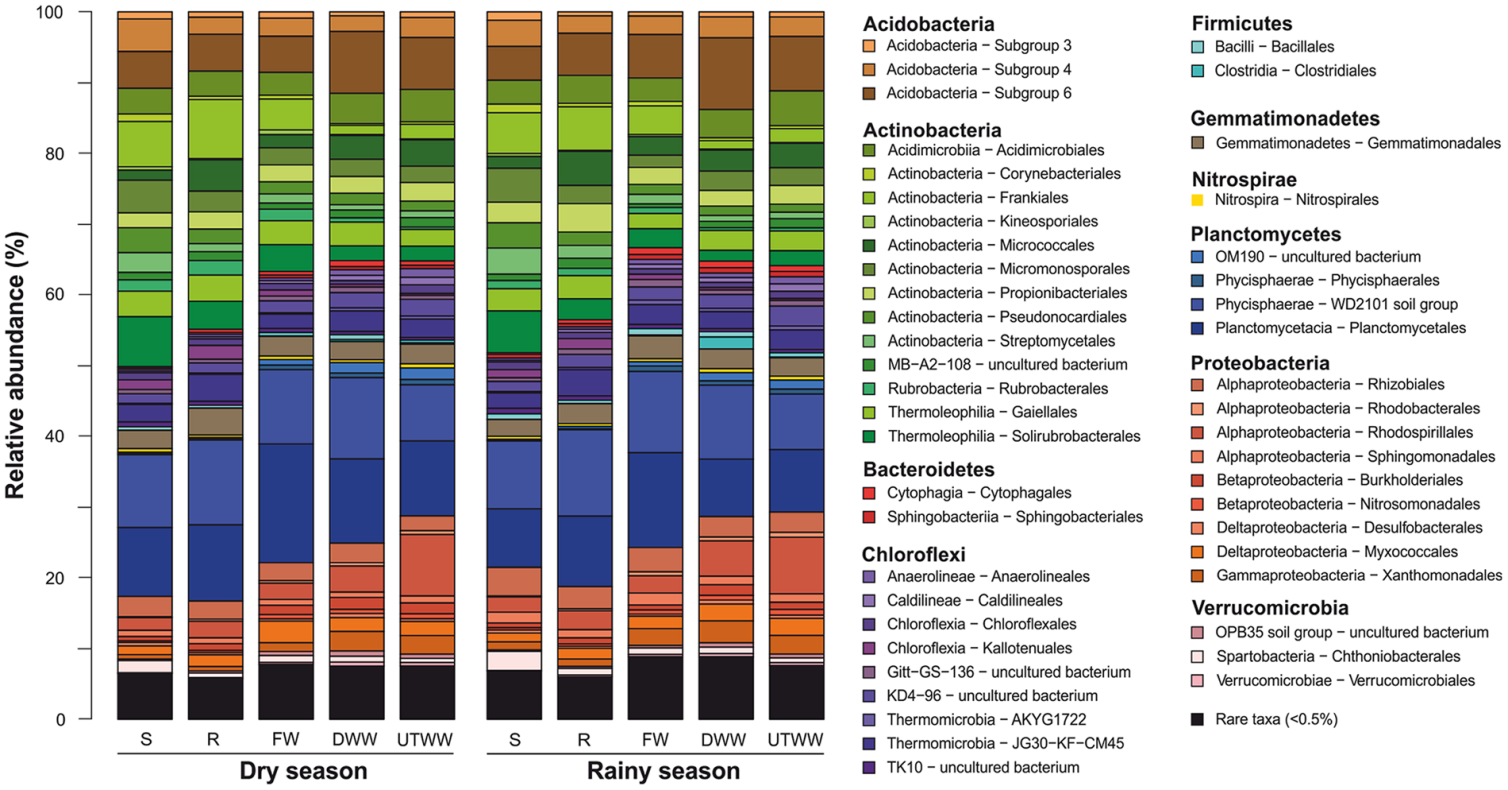
Relative abundances of soil bacterial orders derived from DNA. Land use systems: Shrubland (S), Rainfed (R), Freshwater (FW), Dam wastewater (DWW) and Untreated wastewater (UTWW) irrigated, during dry and rainy season. Bacterial orders with average relative abundances > 0.5% is visualized; orders contributing ≤ 0.5% were summarized as rare taxa.

**Figure 5:**
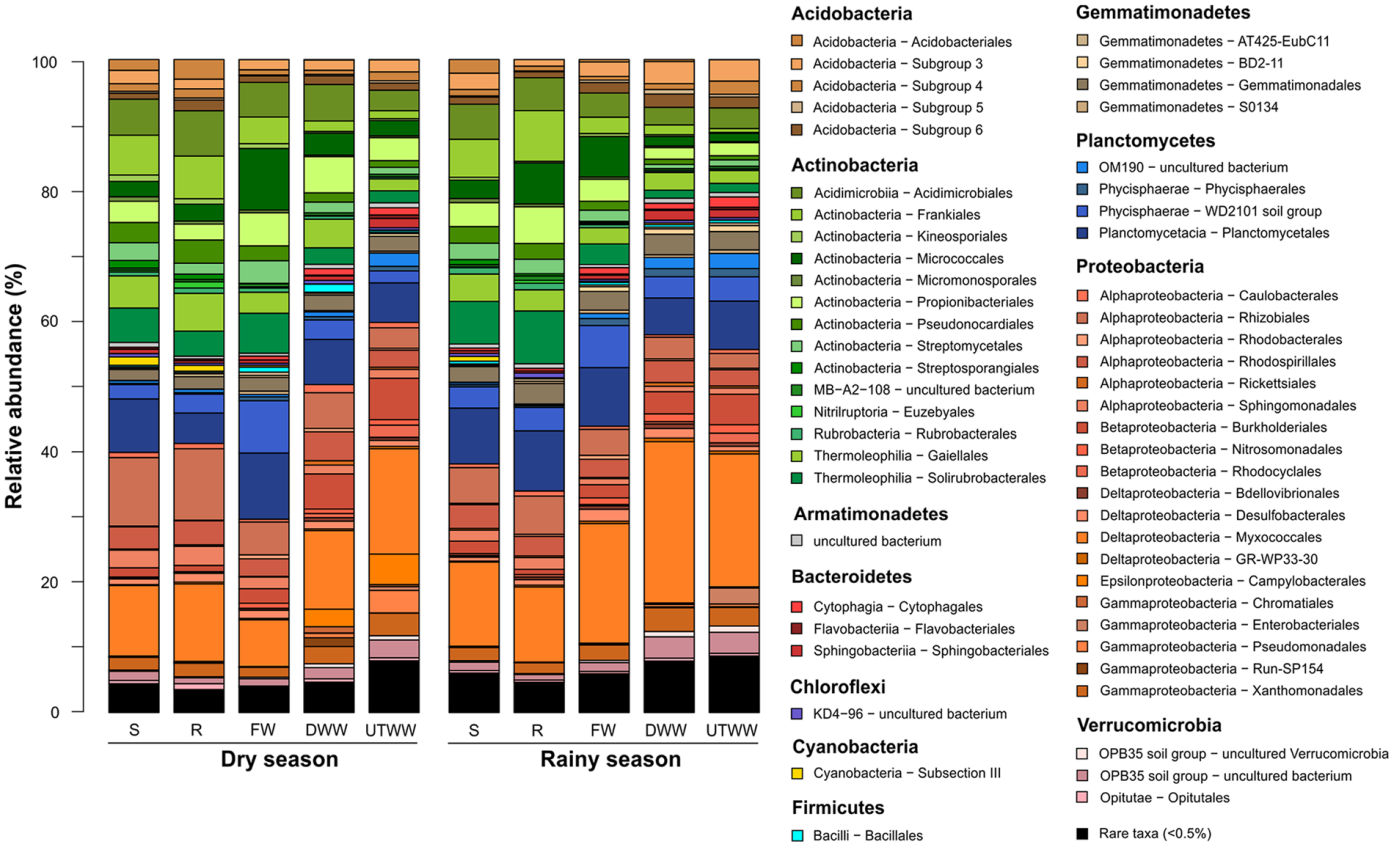
Relative abundances of soil bacterial orders derived from RNA. Land use systems: Shrubland (S), Rainfed (R), Freshwater (FW), Dam wastewater (DWW) and Untreated wastewater (UTWW) irrigated, during dry and rainy season. Bacterial orders with average relative abundances > 0.5% is visualized; orders contributing ≤ 0.5% were summarized as rare taxa.

The OTUs across all samples were assigned to 45 bacterial phyla and more than 390 orders at DNA and RNA level. The dominant phyla (>0.5% of all sequences) were *Actinobacteria* (30% DNA and RNA), *Planctomycetes* (23% DNA, 14% RNA), *Proteobacteria* (16% DNA, 38% RNA), *Acidobacteria* (11% DNA, 5.9% RNA), *Chloroflexi* (11% DNA, 1.4% RNA), *Gemmatimonadetes* (3.4% DNA and RNA), *Verrucomicrobia* (2.3% DNA, 3.3% RNA), *Bacteroidetes* (1.2% DNA, 1.7% RNA), *Firmicutes* (1% DNA, 0.9% RNA) and *Nitrospirae* (1% DNA, 0.5% RNA) (Figs. 4 and 5). This high abundance of *Planctomycetes* (23% DNA, 14% RNA) detected in our samples is in the same range as described for other semiarid regions^5^. At order level *Planctomycetales* (8.7% DNA, 4.1% RNA), candidate group WD2101 which was recently assigned to the order *Tepidisphaerales*^46^ (8.3% DNA, 2.1% RNA) were abundant in both entire and active community. At DNA level also Subgroup 6 (*Acidobacteria*) (5.3%), and *Frankiales* (3.3%) and at RNA level *Myxococcales* (7.9%) and *Rhizobiales* (2.5%) were abundant.

Predominant proteobacterial orders, such as *Caulobacterales*, *Rhizobiales*, *Bdellovibrionales*, *Desulfobacterales* and *Myxococcales*; and the verrucomicrobial order *Opitutales* and group OPB35 showed higher relative abundances at RNA level than at DNA level. *Acidobacteria* (subgroups 4 and 6), *Chloroflexi*, *Nitrospira and Planctomycetes* (candidate group WD2101 and *Planctomycetales*) showed the opposite trend. This is possibly related to the different life strategies of these taxa. *Proteobacteria* show copiotrophic activity and high grow rates when resources are abundant and preference for labile organic C consumption^47^. *Acidobacteria*, *Chloroflexi*, *Nitrospirae* and *Planctomycetes* have been categorized as oligotrophs with slow growth rates and thriving capacity in nutrient-poor environments^26,47,48^. *Verrucomicrobia* follow oligotrophic strategies^49^, however, it is indicated that their water-related opportunistic behaviour^35^ has a strong influence on their activity, as in our study they are more active in wastewater irrigated soil.

### Land use systems shape bacterial community

To identify orders that were significantly associated with one, two, or more land use systems, we performed a correlation-based association analysis. All bacterial orders were included in the analysis, 362 at DNA level and 371 at RNA level. On average 56% of the orders were not significantly different (p ≤ 0.05) in relative abundance with respect to land use system.

**Figure 6:**
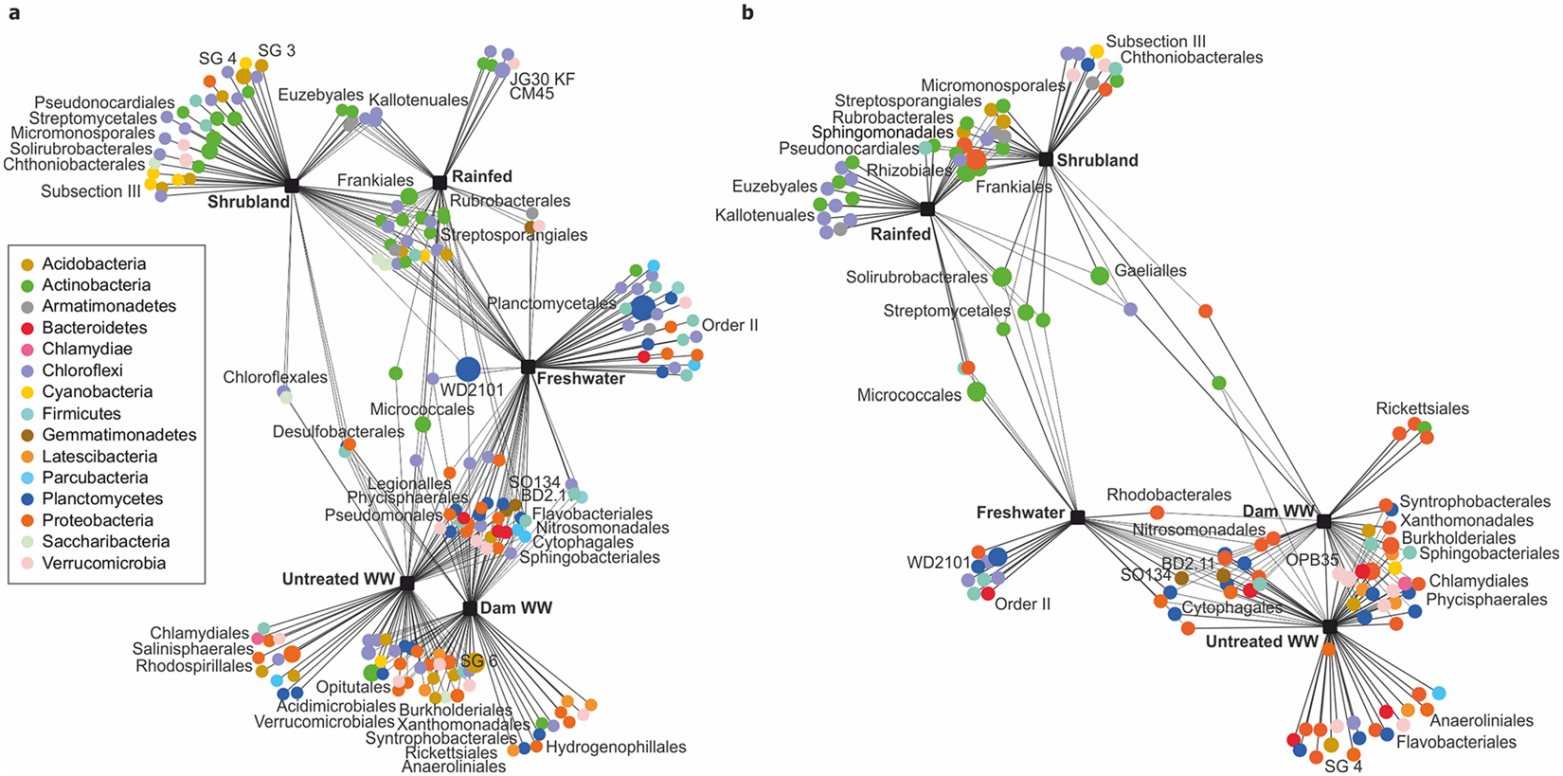
Correlation-based association network at order level of total (**a**) and potentially active (**b**) bacterial communities among land use systems: shrubland, rainfed, freshwater, dam wastewater and untreated wastewater irrigated. Only statistical significant bacterial orders are visualized (p ≤ 0.05). The size of each node is proportional to the taxon relative abundance and the edge width corresponds to the association strength of each taxon with the land use system.

The correlation-based association analysis (Fig. 6) is consistent with the multivariate analysis (Fig. 3). Bacterial communities from shrubland sites and rainfed systems showed similarities, they shared on average 3.2% of bacterial orders. Even more similar were soil communities irrigated with wastewater (DWW and UTWW), they shared 7.5% bacterial orders. The FW clustered between these two groups, sharing 3.8% bacterial orders with the drier systems (shrubland and rainfed) and 5.5% with the systems under monthly irrigation.

The groups *Chthoniobacterales* and subsection III (*Microcoleus*) were associated uniquely to shrubland soil. *Microcoleus* species are desiccation tolerant and photosynthetic and are found in arid soils worldwide^50^. Only two alphaproteobacterial taxa (*Rhizobiales* and *Sphingomonodales*), as well as *Pseudonocardiales*, *Micromonosporales*, *Euzebyales* and *Kallotenuales* were associated to shrubland and rainfed soil. The last two seem to be only active in rainfed soil. *Rhizobiales* and *Sphingomonadales* have previously been associated with arid soils^51^. The wastewater free systems, which are also the driest (shrubland, rainfed and FW) shared 23 bacterial orders, most of them are actinobacterial (*Rubrobacterales*, *Frankiales*, *Pseudonocardiales*, *Streptosporangiales*, *Streptomycetales* and *Solirubrobacterales*). In general, we observed that irrigation and predominantly irrigation with wastewater diminished the abundance of *Actinobacteria*. This phylum is known to harbour drought resistant groups, specifically, class *Rubrobacteridae* increase in abundance during desiccation^35^. Groups associated uniquely to FW were *Planctomycetales* and Order II (*Cytophagia*). *Tepidisphaerales* (formerly WD2101) was one of the most abundant orders but only showed activity in FW. Land use systems under monthly irrigation (FW, DWW and UTWW) shared 27 orders only few of them were active, among those were *Cytophagales*, *Nitrosomonadales* and two *Gemmatimonadetes* candidate groups (SO134 and BD2.11). Finally, the systems under wastewater irrigation shared 30 orders, most of them were active in one or both systems. These orders belonged mainly to *Proteobacteria* (*Xanthomonadales*, *Burkholderiales*, *Synthrophobacterales*, *Anaeroliniales*, *Rickettsiales*) and *Bacteroidetes* (*Sphingobacteriales*, *Flavobacteriales*), as well as, *Chlamydiales* and *Phycisphaerales*. Several of these orders are known for comprising pathogenic representatives. Wastewater clearly transfers members of *Bacteroidetes* and *Proteobacteria* into the soil (Supplementary Material Fig. S6), orders such as *Flavobacteriales*, *Rhodospirillales*, *Burkholderiales*, *Syntrophobacterales* and *Pseudomonadales* were detectable in the corresponding wastewater irrigated soils. *Bacteroidetes* have been shown to be more abundant in agricultural systems than in natural ones, and their abundance is also associated to fertilized soil^52^. The *Xanthomonadaceae* family has been previously reported to exhibit higher abundance in treated wastewater irrigated soil^25^. In general, we observed that irrigation enhanced the abundance of *Planctomycetes*, *Proteobacteria*, *Bacteroidetes* and *Gemmatimonadetes*. Additionally, wastewater irrigation increased the abundance of *Chlamydia*. These results are consistent with previous studies on dryland soil^5^ and on treated^25^ and untreated^19^ wastewater irrigated soils.

Finally, the correlation of diversity indices to soil properties suggests lineage-specific adaptations, bacterial taxa associated to wastewater irrigated soil are adapted to higher concentrations of sodium ions and the corresponding salt stress provided by the wastewater. The taxa associated to wastewater irrigated soil might be also adapted to surfactants and heavy metals present in wastewater^29^, that were not monitored in this study. The bacterial orders associated to shrubland, rainfed and partially FW are adapted to drought, hence sensible to sodium content and probably other contaminants.

### Land use systems and seasonality defines the bacterial functional profile

**Figure 7:**
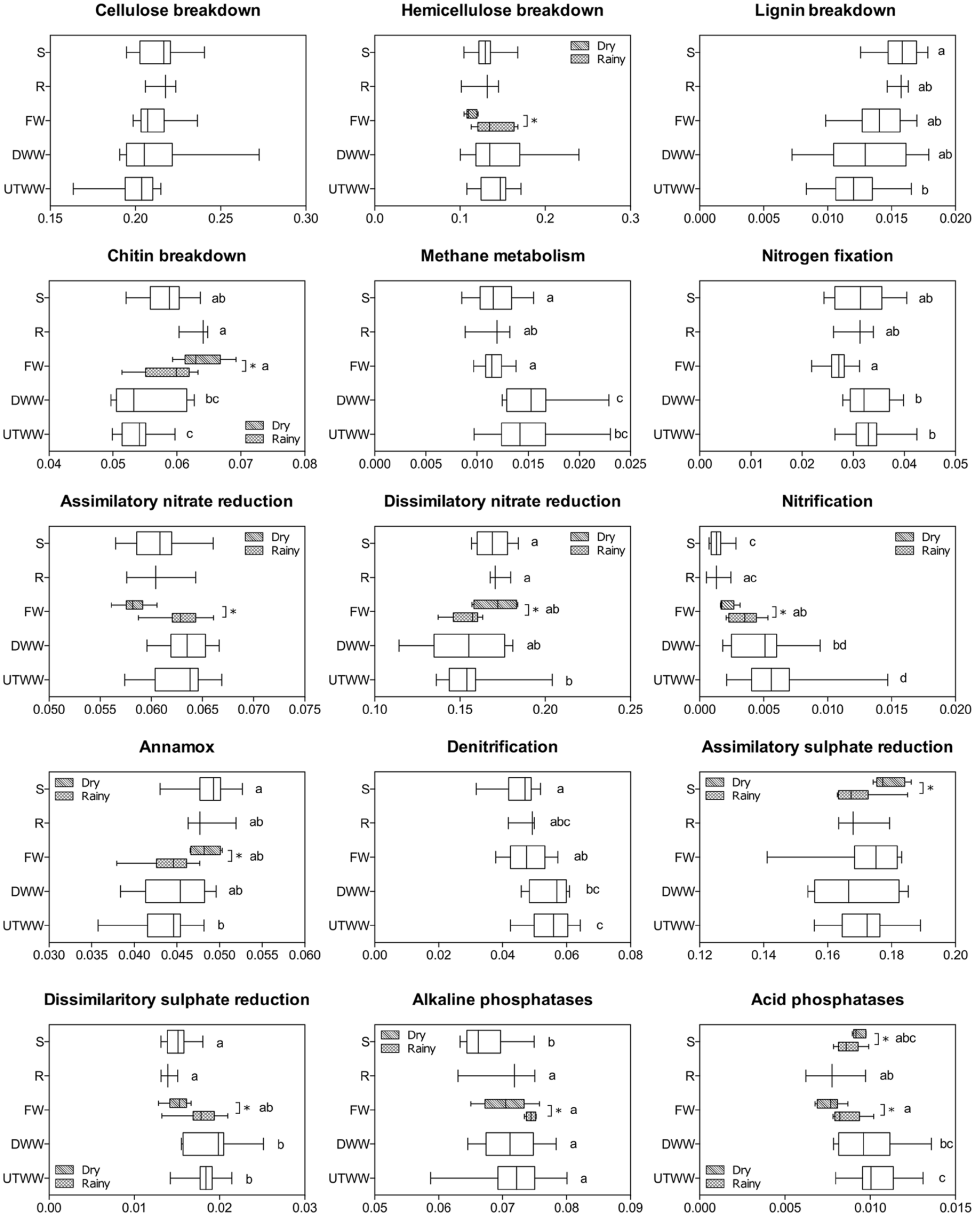
Relative abundances of key enzymes calculated from the potentially active bacterial community in each land use system. Shrubland (S), Rainfed (R), Freshwater (FW), Dam wastewater (DWW), and Untreated wastewater (UTWW) during the dry and rainy season. Key genes were combined and their mean abundance is shown. Box are extended from the 25^th^ to 75^th^ percentiles, the line in the box is plotted at the median. Whiskers represent the smallest and the largest value. Kruskal-Wallis and Dunn’s tests were used to determine differences among land use systems, and permutation test for differences between seasons. Only parameters differing significantly between seasons in each land use system are shown, if they did not differ, samples of both seasons were merged. The enzymes included in the analysis are given in the Supplementary Material Table S3.

Bacterial community functions participate in critical processes needed to maintain soil quality and ecosystem functioning. We predicted the functional capabilities of the potentially active bacterial (RNA-based) communities in relation to the C, N, S, and P cycles by employing Tax4Fun^53^. Abundances of important enzyme-encoding genes participating in different pathways within these cycles differed among land use systems and seasons (p ≤ 0.05; Fig. 7). Genes involved in nitrification and denitrification had higher abundances in wastewater irrigated soil (DWW and UTWW). This effect might be due to the high N supply by the wastewater (ammonium and organic N)^37^, and the higher abundance of genera like *Nitrosospira*, *Nitrosomonas* (*Nitrosomonadales*), *Nitrospira* (*Nitrospirae*), *Nitratireductor* (*Rhizobiales*) and *Massilia* (*Burkholderia*). Friedel and colleagues^29^ reported a higher denitrification capacity in wastewater irrigated soil than in rainfed soil of the Mezquital Valley. Further, nitrous oxide emissions from wastewater irrigated fields cropped with maize are 10-fold larger than from rainfed fields^54^. Methane metabolism genes were also more abundant in soil under wastewater irrigation, this might be associated to the high methane content of wastewater^55^ and the higher abundance of *Verrucomicrobia* and *Gammaproteobacteria* in wastewater irrigated soils, as they comprise methanotrophic taxa, such as *Methylacidiphilum* and *Methylococcacea*, respectively^56^. The abundance of genes related to dissimilatory sulphate reduction was also higher in wastewater irrigated systems, probably due to the higher amount of sulphate present in the untreated wastewater. Genes related to nitrogen fixation were less abundant in FW compared to wastewater irrigated systems, contrary to the general trend that high nitrate contents decrease the efficiency of the N fixation and the unfavourable effects of salinity for nodule development^39^. Furthermore, an increase in genes related to lignin and chitin breakdown was observed in shrubland and rainfed soils, probably caused by the higher abundance of genera like *Arthrobacter* (*Micrococcales*) and *Streptomyces* (*Streptomycetales*), which are known to degrade single ring aromatic substrates^57^, and contain larger numbers of chitinase encoding genes per genome^58^. These results are in accordance with soil organic matter quality analyses performed in the same land use systems^59^, which evidenced a much stronger microbial degradation of lignin in the nutrient-limited shrubland than in wastewater irrigated fields. The abundance of genes related to dissimilatory nitrate reduction and annamox were also higher in shrubland soil and lower in UTWW. Dissimilatory nitrate-reducing bacteria are generally found in zones with low or limited nitrate availability^60^, as semiarid shrubland and rainfed soil. Phosphatase genes (alkaline phosphatases and acid phosphatase) showed lowest abundances in shrubland and in FW, respectively. We assume that this is related to the difference in soil pH, as FW exhibited the highest pH (7.8) and shrubland the lowest pH (7.1).

We observed seasonal effects on the bacterial functional profile between the dry and the rainy season. As shown before, FW and UTWW communities differed significantly in composition between seasons (p ≤ 0.01) and the DWW community showed seasonal differences in diversity. As expected, several pathways (hemicellulose and chitin breakdown, nitrification, dissimilatory nitrate and sulphate reduction, and acid and alkaline phosphatases) were affected in FW suggesting this community is susceptible taxonomically and functionally to environmental changes. However, no seasonal effect was recorded for the functional traits in the soil communities influenced by DWW and UTWW. This suggests that wastewater irrigated soil bacterial communities are functionally redundant. Redundancy concerning functional diversity enables soil bacterial communities to adapt to changing environmental parameters^61^. Our results indicate that land use change and the irrigation with different water quantity and quality does not only influences bacterial composition but also affects their functional capabilities. Each community reveals a different response towards changing parameters, which might have consequences for soil quality and ecosystem functioning.

### Detection of pathogenic bacteria in wastewater irrigated soils

To determine the potential risk on human and plant health^8^ of introducing pathogenic bacteria in soils by using wastewater for crop irrigation, especially untreated wastewater, we analyzed the occurrence of several common bacterial genera containing pathogenic members in soil (DNA and RNA-based) and wastewater samples (DNA-based). The wastewater use for irrigation in the Mezquital Valley harboured known pathogenic bacterial species like *Acinetobacter baumannii*, *A*. *junii*, *A*. *johnsonii*, *A*. *lwoffii*, *Bacillus thuringiensis/cereus/anthracis*, *Citrobacter freundii*, *Clostridium perfringens*, *Corynebacterium ulcerans*, *Enterococcus faecium*, *Klebsiella oxytoca*, *Moraxella canis*, *M*. *osloensis*, *Mycobacterium cosmeticum*, *M*. *terrae*, *Shigella dysenteriae/flexneri/sonnei*, *Staphylococcus hominis*, *Streptococcus anginosus*, *S*. *lutetiensis*, and *S*. *pneumonia*. *Shigella dysenteriae/flexneri/sonnei* and *A*. *johnsonii* were the most abundant bacterial OTUs in the wastewater samples (Supplementary Material Table S4). Different species of these genera are commonly present in wastewater and are considered waterborne pathogens^62,63^. In the soils of the Mezquital Valley we found eight species with an increased abundance in the DWW and UTWW (p ≤ 0.05) and for seven of them activity was indicated by occurrence at RNA level: *Acinetobacter baumanni*, *A*. *soli*, *A*. *junii*, *A*. *haemolytic*, *A*. *schindleri*, *Bacillus thuringiensis/anthracis/cereus and Nocardia flavorosea* (Supplementary Material Fig. S7). These microorganisms have been previously reported in connection with treatment and use of wastewater for irrigation^63–65^. *B*. *anthracis*, *B*. *cereus*, *and B*. *thuringiensis* are members of the Bacillus cereus group; *B*. *anthracis* causes the fatal disease anthrax, *B*. *thuringiensis* produces toxic proteins to some insect larvae, therefore it is commonly used as a biological pesticide. *B*. *cereus* is an ubiquitous soil bacterium and an opportunistic pathogen, which commonly causes food poisoning^66^. *Acinetobacter* and *Nocardia* species are frequently found in soil, water and decaying fecal deposits, and are recognized as opportunistic pathogens mainly in immunocompromised patients^67,68^. In the wastewater irrigated soil of the Mezquital Valley, Broszat and colleagues^19^ obtained isolates of *Acinetobacter* and *Bacillus spp*. resistant to antibiotics. Our results indicate that only few waterborne pathogens survive under soil conditions, and *Acinetobacter*, *Bacillus* and *Norcadia spp*. are in low abundances, yet they are potentially active and harmful, representing a potential health issue mainly in Mezquital Valley areas were wastewater is used for crop irrigation.

The phytopathogens *Acidovorax valerianellae* and *Rhizorhabdus argentea* were detected in the inlet wastewater; species of these genera have been previously found in wastewater treatment plants^8^. Within soil, we detected nine species of plant pathogens from the genera *Pseudomonas*, *Streptomyces*, *Pantoea*, *Ralstonia* and *Rhodococcus*, from which only *R*. *fascians* was potentially active. *Rhodococcus fascians* provokes abnormal growth in a broad range of plants^69^. Members of the genera *Pseudomonas*, *Ralstonia* and *Xanthomonas* have been previously detected in irrigation systems^70^. These results suggest that wastewater irrigation of crops can have detrimental effects on the growth of specific plant species. However, in this area the main crops are maize and alfalfa, which are not targeted by the detected phytopathogens.

## Conclusion

In line with our general hypothesis dryland transformation to agricultural land under irrigation with different water quantity and quality exhibits a significant effect on the soil bacterial composition, diversity and functionality at entire and active community level. In agreement with our hypothesis (b), we observed shrubland and rainfed soil bacterial communities had similar structure and even closer was the structure of the dam and untreated wastewater irrigated soil communities. The structure of the freshwater irrigated soil shares taxa with both, the driest and the wastewater irrigated soil, as an intermediate land use systems. In contrast to our hypothesis (c), the bacterial diversity of the natural dryland community was only higher compared to that from the rainfed system but not to those of the other systems. In accordance with our hypothesis (a) soil moisture in addition with sodium content and soil pH were the strongest drivers of bacterial community structure. Our results indicate lineage-specific adaptations to drought and sensitivity to sodium content for taxa associated to shrubland, rainfed and freshwater irrigated soil; taxa associated to freshwater, dam wastewater and untreated irrigated systems show the opposite trend. The seasonal difference in the community structure was only evident at active community level in freshwater and untreated wastewater irrigated systems. The freshwater-irrigated soil communities appear sensitive to environmental changes since their composition and functionality are severely affected. The soil community under wastewater irrigated systems varies in diversity and composition, but it seems that their high functional redundancy favours them to resist changing conditions as their functionality is not altered. These emphasizes the advantage of using rRNA-based analyzes to evaluate short term effects on the soil bacterial community. We could identify potentially harmful human and phytopathogens that might be a health risk for the population but not for the predominant crops in the area. In the future, we will focus on the analysis of seasonal changes of the soil microbial community structures and their functions along the different land use systems in drylands. Interactions between different prokaryotic groups and other soil microorganisms including fungi will be investigated to expand our understanding of how dryland transformation influences soil quality and ecosystem functions.

## Methods

### Study sites and sample collection

This survey was performed in the Mezquital Valley (Fig. 1), located 100 km north of Mexico City in the state of Hidalgo (20°7′44′′N and 99°12′54′′O). The valley has a semi-arid climate with average annual temperatures from 16 to 18 °C and an average annual rainfall from 400 to 600 mm^71^. The natural vegetation in the area is classified as xerophytic shrubs with mesquite (*Prosopis juliflora*) as the dominant tree species. Shrubland areas are located in the mountains and upper piedmont areas, where no irrigation infrastructure exists. The main crop is maize (*Zea mays*), which is produced in the rainy season (June-October), or alfalfa (*Medicago sativa*), a perennial fodder crop under irrigated agriculture. Alfalfa is rotated with maize (three years of alfalfa and two years of maize). The fields under rainfed agriculture are abandoned the rest of the year. The lowest areas of the valley are irrigated periodically (every 30 days on average) with fresh water pumped from deep wells, untreated wastewater coming from Mexico City or wastewater temporally stored in the Endhó dam where a sedimentation process occurs during 3 months of storage. Irrigation is performed by flooding, with 200 mm per irrigation^71^. Alfalfa receives 10 irrigations per year and maize 6 irrigations during the growing cycle.

The sampling included four plots × five land use systems × four samplings = 80 soil samples. The land use systems included sites with natural dryland vegetation (shrubland plots S1–S4), rainfed plots (RF1 to RF4), plots irrigated with freshwater (FW1 to FW4), plots irrigated with wastewater coming from the Endhó dam (DWW1 to DWW4) and plots irrigated with untreated wastewater (UTWW1 to UTWW4) (Fig. 1). The size of the plots ranged among one to two ha. The sampling was performed four times in the same 20 plots, twice during the rainy season (June - October) 2014, and twice during the dry season (November - May) in 2015). The plots used for agriculture were cropped with maize during the rainy season, during the dry season the plots were fallow or cropped with alfalfa, oat or grass (irrigated sites). At each sampling plot, 20 bulk soil cores (6 cm diameter) from the upper 10 cm were sampled in a regular systematic grid; the cores were homogenized and pooled into one composite sample per plot. Plant debris and stones were removed from the homogenized composite samples. One part of the soil samples was frozen in liquid nitrogen and kept at −80 °C and the other part was air dried for soil characterization.

### Characterization of soil parameters

The soils of all plots were classified as Haplic Phaeozem^72^ (IUSS, 2014) with clay to silty clay loam texture. The soil samples were air-dried for 24 h, homogenized and sieved using a metallic mesh (2 mm). The physical and chemical properties were determined using standard procedures^73^. The soil pH was measured in a 1:2 soil:CaCl_2_ 10 mM suspension using a sensION156 HACH pH meter (Conductronic pH 120, Puebla, Mexico) and the electric conductivity was determined in a 1:2 soil:distilled water suspension with a conductivity meter (Hanna HL4321, Rhode Island, USA). Soil organic C content and total N was determined using a Perkin Elmer 2400 CHNS/O elemental analyzer (Massachusetts, USA). Available P was determined by spectrophotometry (Genesis 20, Massachusetts, USA) based on the methodology developed by Olsen^74^. Soil particle size distribution was determined using the Bouyoucos hydrometer method. Exchangeable base cations (Ca^2+^, Mg^2+^, Na^+^ and K^+^) were extracted with a 1 N solution of ammonium acetate (NH_4_OAc) at pH 7, and the determination of Ca^2+^ and Mg^2+^ was done by atomic absorption spectrophotometry (Perkin Elmer 3110, Massachusetts, USA), and the one of Na^+^ and K^+^ by flame emission spectrometry (Sherwood Scientific 36, Cambridge, UK), according to van Reeuwijk (1992)^74^. The gravimetric soil water content (%) was calculated from oven-dried subsamples. The soil properties and crop type of each sample and plot are shown in Table S1 of the Supplementary Material.

### Nucleic acid extraction, cDNA synthesis and amplification of 16 S rRNA genes and transcripts

Soil samples for microbiological analyses were freeze-dried with liquid nitrogen at the field, they lasted 3–4 days in liquid nitrogen (during field campaign) and were then transferred to −80 °C, where they remained until nucleic acid extraction. DNA was extracted from approximately 0.25 g soil by employing the MoBio PowerSoil DNA isolation kit (MoBio Laboratories, Carlsbad, CA, USA) following the instructions of the manufacturer. Total RNA was extracted from approximately 2 g of soil per sample by employing the PowerSoil total RNA isolation kit (MoBio Laboratories, Carlsbad, CA, USA) according to the manufacturer’s instruction. Nucleic acid concentrations were quantified using a NanoDrop ND-1000 Spectrophotometer (Thermo Scientific, Schwerte, Germany). Subsequently, total RNA extracts were treated with Turbo DNase to remove remaining DNA and purified by using the Qiagen RNeasy MinElute Cleanup kit (Qiagen, Hilden, Germany). The presence of remaining DNA was tested by PCR as described by Wemheuer *et al*.^75^. Purified RNA (30–100 ng) was converted to cDNA by using the SuperScript III as recommended by the manufacturer (Thermo Scientific, Schwerte, Germany). Bacterial 16S rRNA gene and transcript amplicons were generated using the bacterial primers targeting the V3-V4 region described by Klindworth *et al*.^76^ with adapters for Illumina MiSeq sequencing. The PCR reaction mixture (25 µl) contained 5-fold Phusion GC buffer, 200 µM of each of the four deoxynucleoside triphosphates, 5% DMSO, 0.4 µM of each primer, 1 U of Phusion HF DNA polymerase (Fisher Scientific GmbH, Schwerte, Germany), and 25 ng of isolated DNA or cDNA as template. For DNA amplification, the following cycling scheme was used: initial denaturation at 98 °C for 5 min and 25 cycles of denaturation at 98 °C for 45 s, annealing at 60 °C for 30 s and extension at 72 °C for 30 s, followed by a final extension at 72 °C for 10 min. PCR reactions were performed in triplicate for each sample. The resulting PCR products were pooled in equal amounts and purified using the QIAquick Gel Extraction kit (Qiagen, Hilden, Germany) as recommended by the manufacturer. Quantification of the PCR products was performed using the Quant-iT dsDNA HS assay kit and a Qubit fluorometer as recommended by the manufacturer (Invitrogen GmbH, Karlsruhe, Germany). Indexing of the PCR products was performed with Nextera XT Index kit as described by the supplier (Illumina, San Diego, CA, USA). Sequencing of 16 S rRNA was performed using the dual index paired-end approach (2 × 300 bp) with v3 chemistry for the Illumina MiSeq platform.

### Sequence processing and analyses

Demultiplexing of raw sequences was performed by CASAVA data analysis software (Illumina). Paired-end sequences were merged using PEAR v0.9.10 (64 bit) with default parameters^77^. Sequences with average quality score lower than 20 or containing unresolved nucleotides were removed from the dataset with the *split_libraries_fastq*.*py* script from QIIME 1.9.1^78^. We additionally removed remaining unclipped reverse and forward primer sequences by employing cutadapt v1.10^79^ with default settings. For operational taxonomic unit (OTU) clustering, we used USEARCH (8.1.1861) with the UPARSE algorithm^80^ to dereplicate, remove singletons, and sort all quality-filtered sequences by length (400 bp). Subsequently, OTUs were clustered at 97% sequence identity using USEARCH. Chimeric sequences were removed using UCHIME^81^ in reference mode against RDP trainset15_092015.fasta (https://sourceforge.net/projects/rdp-classifier/files/RDP_Classifier_TrainingData/). All quality-filtered sequences were mapped to chimera-free OTUs and an OTU table was created using USEARCH. Taxonomic classification of the picked reference sequences (OTUs) was performed with *parallel_assign_taxonomy_blast*.*py* against the SILVA SSU database release 123.1^82^. Extrinsic domain OTUs, chloroplasts, and unclassified OTUs were removed from the dataset by employing *filter_otu_table*.*py*. Sample comparisons were performed at the same surveying effort, utilizing the lowest number of sequences by random selection (DNA level 18,900 and RNA level 19,600). Species richness, alpha and beta diversity estimates and rarefaction curves were determined using the QIIME 1.9.1 script *alpha_rarefaction*.*py*.

### Prediction of bacterial functional profiles

Functional profiles were predicted from obtained 16S rRNA data using the software package Tax4Fun^53^. Genes encoding key enzymes involved in nutrient cycles were identified in the resulting profiles using their KEGG orthologs (Supplementary Material Table S3). Mean abundances of genes in each land use system were used for statistical analyses (relative to mean abundance in the complete datasets).

### Identification of human- and phyto-pathogens

We searched the rarefied soil and wastewater OTU tables for genera containing potentially human pathogenic or phytopathogenic members such as *Enterococcus*, *Staphylococcus*, *Klebsiella*, *Acinetobacter*, *Pseudomonas*, *Enterobacter*, *Escherichia*, *Salmonella*, *Campylobacter*, *Vibrio*, *Shigella*, *Clostridium*, *Bacillus*, *Yersinia*, *Helicobacter*, *Mycobacterium*, *Legionella*, *Peptostreptococcus*, *Corynebacterium*, *Streptococcus*, *Neisseria*, *Bartonellas*, *Serratia*, *Bordetella*, *Citrobacter*, *Proteus*, *Treponema*, *Brucella*, *Mycoplasma*, *Chlamydia*, *Ureaplasma*, *Rickettsia*, *Listeria*, *Nocardia*, *Streptomyces*, *Actinobacillus*, *Pasteurella*, *Plesiomonas*, *Moraxella*, *Haemophilus*, *Leptospira*, *Borrelia*, *Ehrlichia*, *Anaplasma* and the phytopathogenic genera *Ralstonia*, *Agrobacterium*, *Erwinia*, *Xylella*, *Dickeya*, *Pectobacterium*, *Clavibacter*, *Liberibacter*, *Tatumella*, *Acidovorax*, *Pantoea*, *Serratia*, *Sphingomonas*, *Rhizobacter*, *Rhizomonas*, *Xylophilus*, *Clostridium*, *Clavibacter*, *Arthrobacter*, *Leifsonia*, *Rhodococcus*, *Streptomyces*, *Pseudomonas*, *Xanthomonas*, and *Herbaspirillum* using QIIME 1.9.1 script *filter_taxa_from_otu_table*.*py*. The corresponding sequences were double checked against the nucleotide collection of the NCBI using BLASTn. The OTUs that exhibited > 98% identities to known members of the genus were further analyzed. The pathogenic nature of the thereby identified taxa was confirmed by literature searches. Taxa for which no pathogenic features were described were discarded.

### Statistical analyses

All statistical analyses were conducted employing R version 3.3.1^83^. The results of all statistical tests were regarded significant with p ≤ 0.05. For all statistical analysis, the OTU table clustered at 97% sequence identity was used. The statistical analyses employed depended on the normality of the data in each variable. For those variables that followed a normal distribution we used ANOVA followed by Tukey’s multiple comparison to test for differences among land use systems and T-test for differences between seasons (bacterial diversity and richness). For those variables following a non-normal distribution we used Kruskal-Wallis analysis of variance followed by Dunn’s multiple comparison to test for differences among land use systems and permutation test (package “perm”)^84^ for differences between seasons (soil properties, potential bacterial pathogens and key genes). To evaluate the diversity indexes relation with the soil parameters Spearman’s rank correlation test was employed (r_s_). To identify the bacterial orders associated with the different land use systems, an analysis based on the point biserial correlation coeficient was performed using *multipatt* (package “indicSpecies”)^85^. For visualization, a network was generated using the land use systems as source nodes, and the bacterial orders as target nodes. All taxa with significant (p ≤ 0.05) associations were visualized in the networks. Network generation was performed using the *edge-weighted spring embedded layout* algorithm in *Cytoscape*^86^, with the edge weight corresponding to the association strength of each order with each land use system. To assess the (dis) similarity of bacterial communities between land use systems and season ANOSIM test was performed in Qiime^78^ on weighted Unifrac distances^87^. To visualize the multivariate dispersion of community composition a non-metric multidimensional scaling (NMDS) was performed, standard deviation ellipses by land use system were projected onto the ordination, utilizing the function *ordiellipse*. The effects of environmental parameters on the bacterial community were analyzed using the *envfit* function and projected into the ordination with arrows. *Ordiellipse* and *evfit* functions are contained in the “vegan” package^88^.

### Sequence data deposition

The 16S rRNA gene and transcript sequences were deposited in the National Centre for Biotechnology Information (NCBI) Sequence Read Archive (SRA) under bioproject number PRJNA386070.

## Electronic supplementary material

Supplementary Material

Supplementary Table S1
