# Supplementary material for: Drylands soil bacterial community is affected by land use change and different irrigation practices in the Mezquital Valley, Mexico
Source: Sci Rep. 2018 Jan 23;8:1413. doi: 10.1038/s41598-018-19743-x (PMC5780513; doi:10.1038/s41598-018-19743-x)
Supplement: Supplementary file 1 — Supplementary Material [file 41598_2018_19743_MOESM1_ESM.pdf]

Supplementary material

Drylands soil bacterial community is affected by land use change and different irrigation practices in the Mezquital Valley, Mexico

Kathia Lüneberg[1*], Dominik Schneider[2], Christina Siebe[1], Rolf Daniel[2]

* **Corresponding**: KL (kluneberg@geologia.unam.mx)

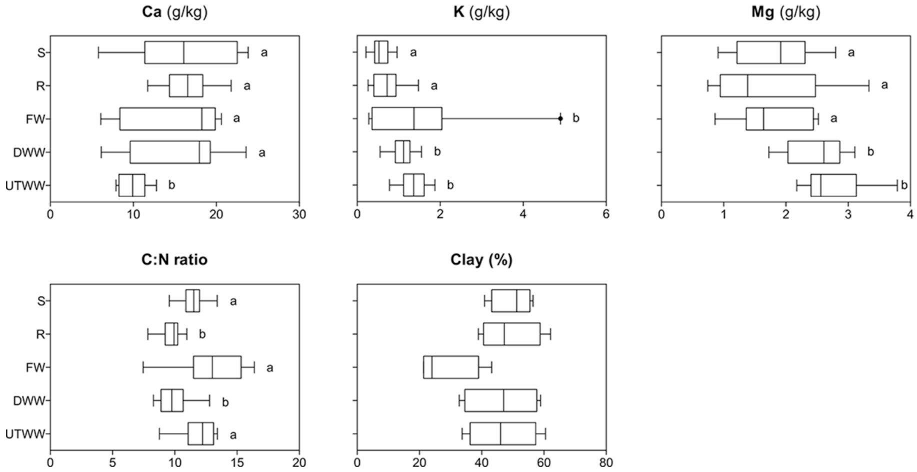

Figure S1. Soil properties in Shrubland (S), Rainfed (R), Freshwater (FW), Dam wastewater (DWW) and Untreated wastewater (UTWW) systems, during the dry and rainy season. Box are extended from the 25th to 75th percentiles, the line in the box is plotted at the median. Whiskers represent the smallest and the largest value. Kruskal-Wallis and Dunn's tests were used to determined differences among land use systems, and permutation test for differences between seasons. Only parameters differing significantly between seasons in each land use system are shown, if they did not differ, samples of both seasons were merged.

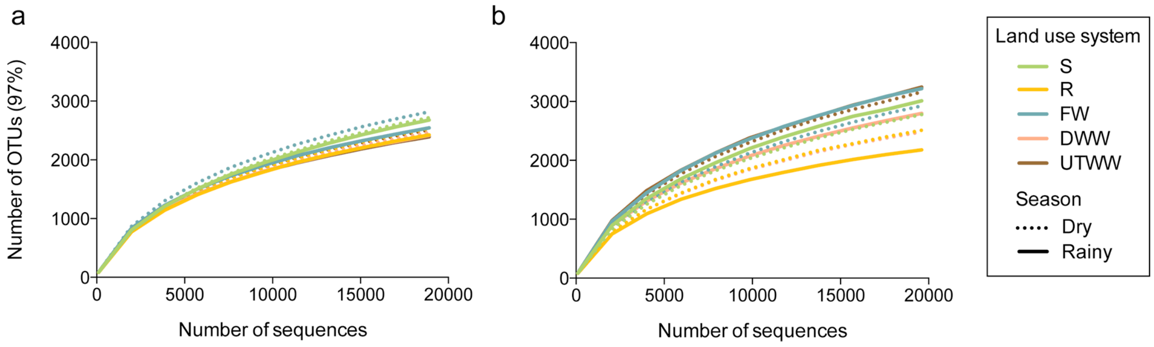

Figure S2. Rarefaction curves at 3% genetic distance from the (a) DNA dataset and the (b) RNA dataset in Shrubland (S), Rainfed (R), Freshwater (FW), Dam wastewater (DWW) and Untreated wastewater (UTWW) systems, during the dry and rainy season. Rarefaction was calculated for a subset of 18,900 sequences per sample for the DNA dataset, and 19,600 sequences per sample for the RNA dataset, in order to prevent artificial overestimation of OTUs based on higher sequence numbers.

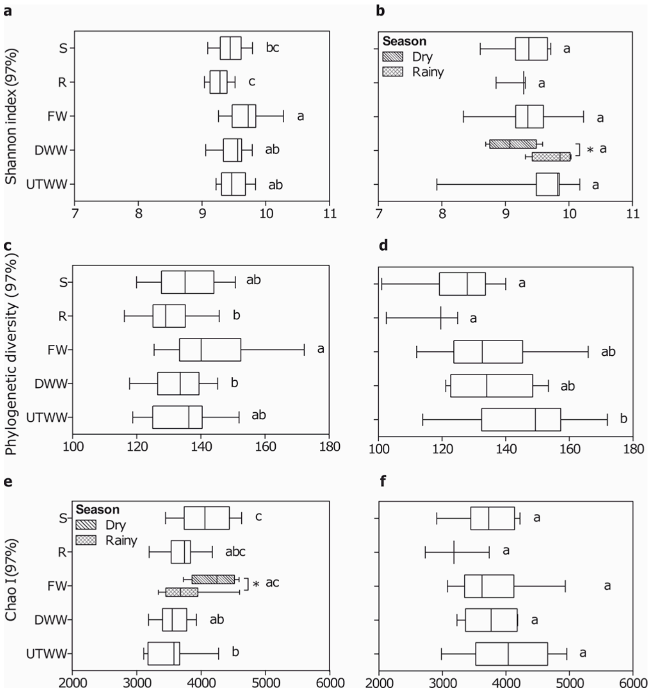

Figure S3. Richness and diversity indices of the total (a, c, e) and the potentially active (b, d, f) bacterial communities in Shrubland (S), Rainfed (R), Freshwater (FW), Dam wastewater (DWW) and Untreated wastewater (UTWW) irrigation systems, during the dry and rainy season. Box are extended from the 25th to 75th percentiles, the line in the box is plotted at the median. Whiskers represent the smallest and the largest value. ANOVA followed by Tukey's multiple comparison tests was used to determine differences among land use systems and the T-test for differences between seasons. Only indices differing significantly ($p \leq 0.05$) between seasons in each land use system are shown separately.

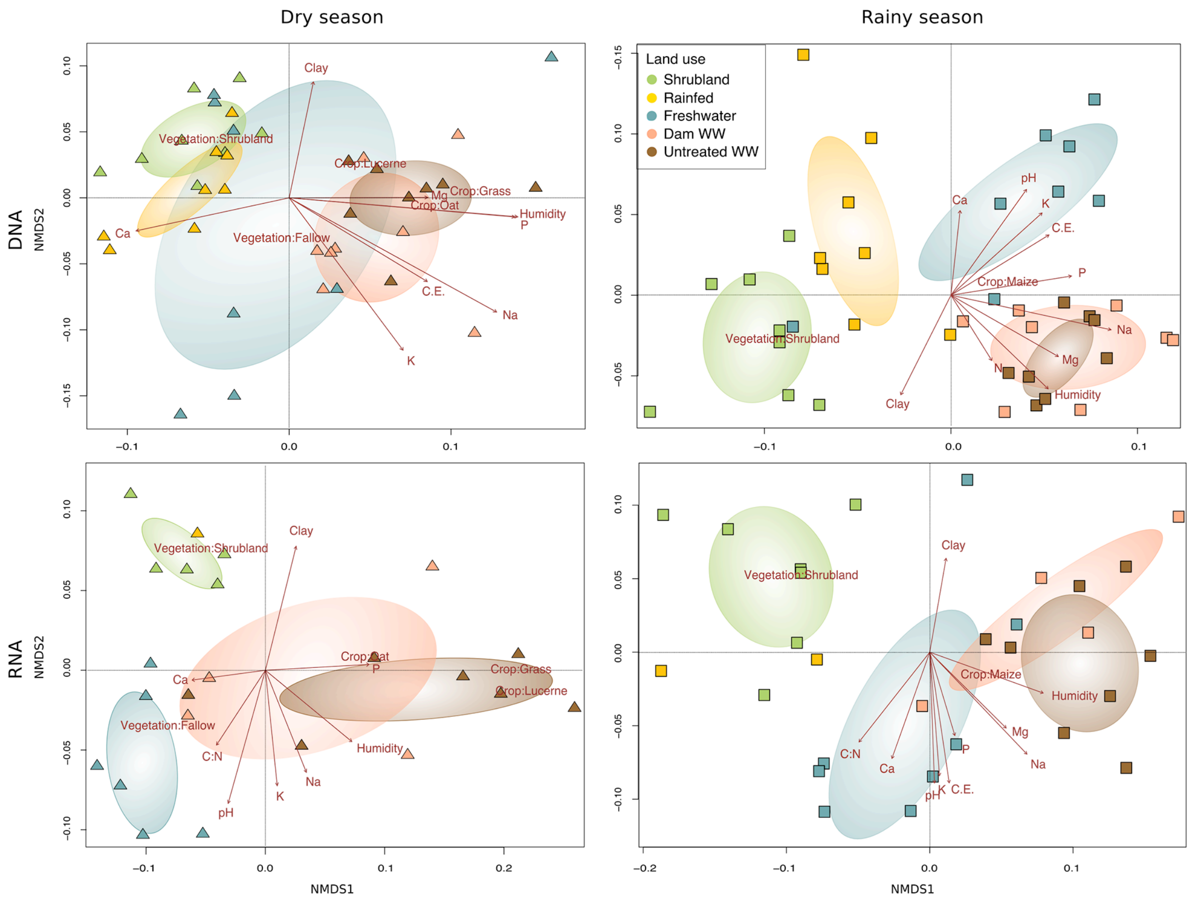

Figure S4. Non-metric multidimensional scaling (NMDS) of total (DNA) and potentially active (RNA) bacterial community composition of samples from the dry and rainy season. Land use systems: shrubland, rainfed, freshwater, dam wastewater and untreated wastewater irrigation based on weighted Unifrac[87] distance matrices. Environmental parameters that were significantly correlated ($p \leq 0.05$) to bacterial community structure are indicated.

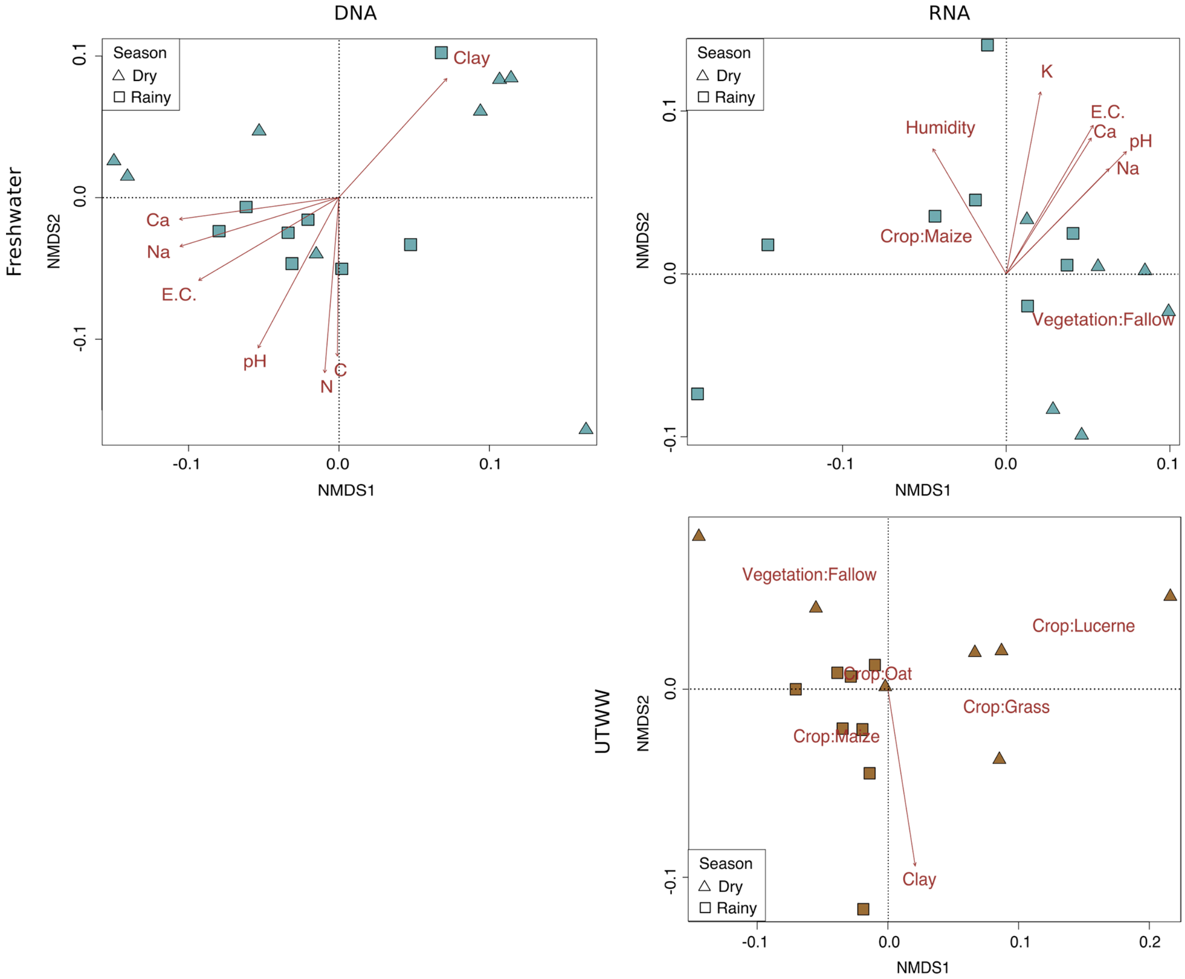

Figure S5. Non-metric multidimensional scaling (NMDS) of total (DNA) and potentially active (RNA) bacterial community composition of freshwater and untreated wastewater irrigation systems based on weighted Unifrac[87] distance matrices. Environmental parameters that were significantly correlated ($p \leq 0.05$) to bacterial community structure are indicated.

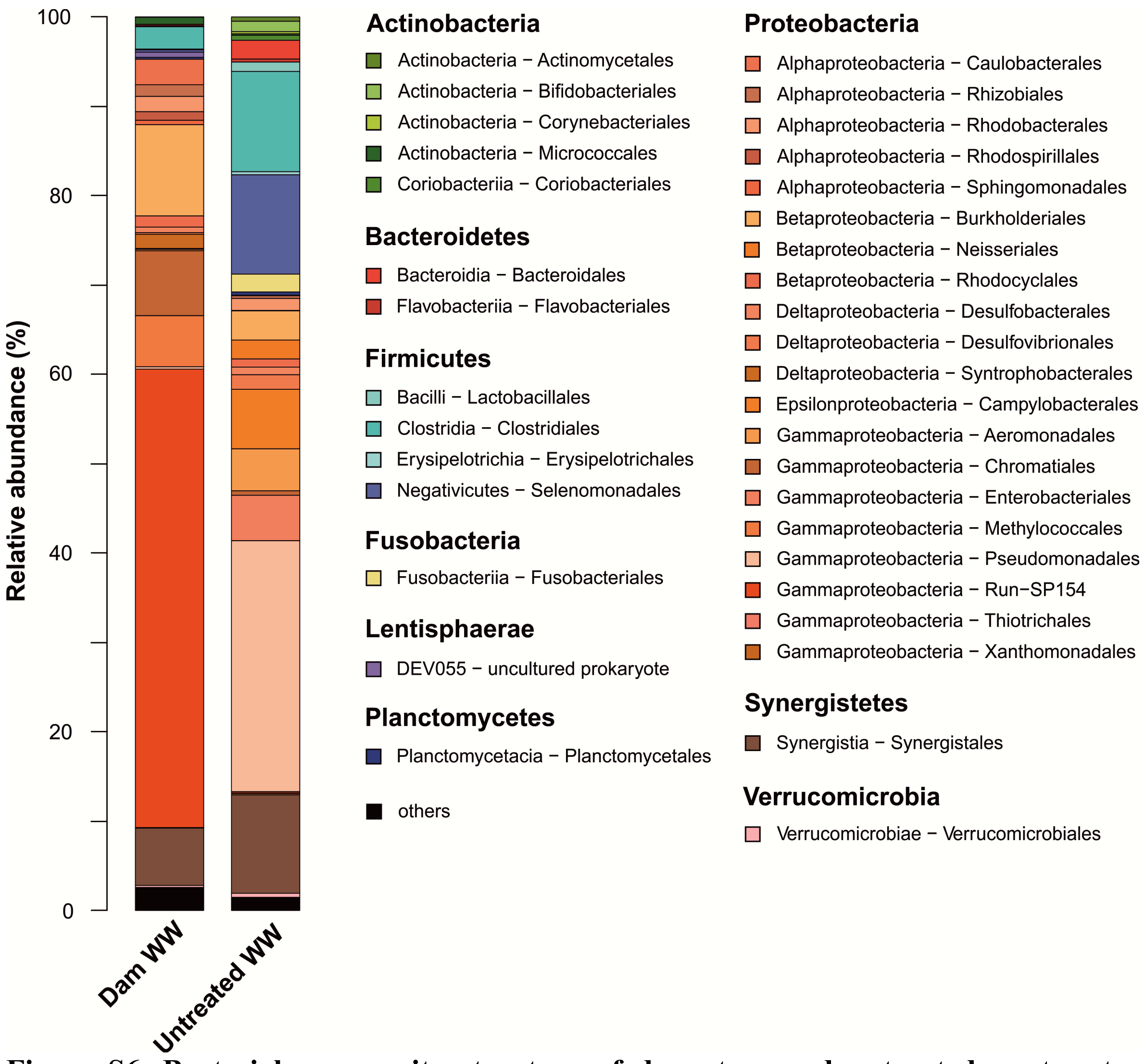

Figure S6. Bacterial community structure of dam-store and untreated wastewater (WW) samples. Mean abundances of the most abundant bacterial orders (> 0.5%) are given. Others: sum of bacterial orders contributing < 0.5%. The number of sequences per sample ranged from 19,404 to 48,931, after rarefaction with the minimal amount of sequences we obtained an average of 374 $\pm$ 15 OTU's per sample.

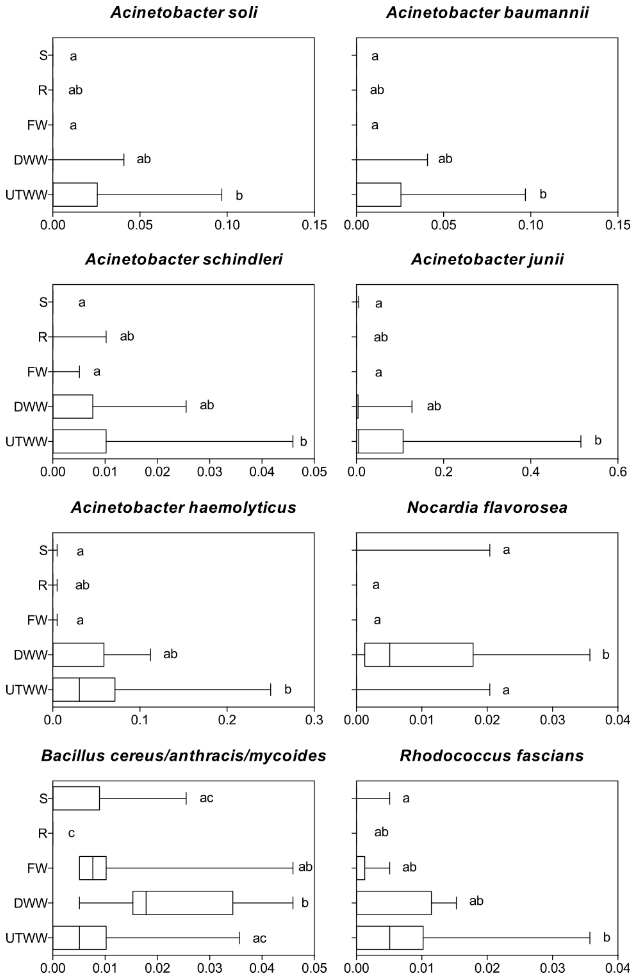

Figure S7. Relative abundance of potentially active pathogens in Shrubland (S), Rainfed (R), Freshwater (FW), Dam wastewater (DWW) and Untreated wastewater (UTWW) systems. Box are extended from the 25th to 75th percentiles, the line in the box is plotted at the median. Whiskers represent the smallest and the largest value. Kruskal-Wallis and Dunn's tests were used to determined differences among land use systems.

Table S2. Spearman correlation coefficient between diversity metrics and soil properties

	DNA			RNA		
	Chao	Shannon	PD	Chao	Shannon	PD
Humidity (%)	-0.12	-0.22	-0.18	0.24	**0.36**	**0.37**
pH	-0.21	**0.34**	0.18	0.08	0.09	0.19
E.C.	0.03	0.03	-0.01	0.13	0.03	0.18
Total Carbon (mg/g)	**0.25**	0.09	0.06	0.11	0.00	0.02
Total Nitrogen (mg/g)	**0.26**	0.03	0.01	0.19	0.05	0.11
C:N ratio	**0.27**	0.06	0.09	-0.14	-0.14	-0.21
P (mg/kg)	-0.16	**-0.24**	**-0.25**	0.16	0.19	0.27
Ca (g/kg)	0.03	**0.33**	0.21	-0.05	-0.07	-0.10
Mg (g/kg)	0.01	-0.07	-0.05	0.12	0.22	0.25
K (g/kg)	-0.18	-0.01	-0.10	-0.03	0.05	0.17
Na (g/kg)	-0.18	-0.22	**-0.22**	0.23	0.26	**0.42**
Clay (%)	0.11	-0.16	-0.04	0.08	0.11	0.03

Bold numbers indicate significance ($p \leq 0.05$). E.C, electrical conductivity; P, available Phosphorous; Ca, Calcium; Mg, Magnesium; K, Potassium; Na, Sodium.

Table S3. Key enzymes of C, N, S, P cycles predicted by Tax4fun

	Key enzyme	Reference
**Carbon cycle**		
Cellulose breakdown	K01179; endoglucanase<br>K01180; endo-1,3(4)-beta-glucanase<br>K01195; beta-glucuronidase<br>K01225; cellulose 1,4-beta-cellobiosidase<br>K05349; beta-glucosidase<br>K05350; beta-glucosidase<br>K16213; mannobiose 2-epimerase	[1], [2]
Hemicellulose breakdown	K01181; endo-1,4-beta-xylanase<br>K01198; xylan 1,4-beta-xylosidase<br>K01811; alpha-D-xyloside xylohydrolase<br>K01218; mannan endo-1,4-beta-mannosidase<br>K01224; arabinogalactan endo-1,4-beta-galactosidase<br>K01684; galactonate dehydratase<br>K01811; alpha-D-xyloside xylohydrolase<br>K15531; oligosaccharide reducing-end xylanase<br>K15921; arabinoxylan arabinofuranohydrolase<br>K15924; glucuronoarabinoxylan endo-1,4-beta-xylanase	[1], [3]
Lignine breakdown	K03381; catechol 1,2-dioxygenase<br>K08689; biphenyl 2,3-dioxygenase subunit alpha<br>K14582; cis-1,2-dihydro-1,2-dihydroxynaphthalene/ dibenzothiophene dihydrodiol dehydrogenase<br>K15750; biphenyl 2,3-dioxygenase subunit beta<br>K14579; naphthalene 1,2-dioxygenase subunit alpha<br>K14580; naphthalene 1,2-dioxygenase subunit beta<br>K00446; catechol 2,3-dioxygenase	[1]
Chitin breakdown	K01183; chitinase<br>K01452; chitin deacetylase<br>K03791; putative chitinase<br>K03933; chitin-binding protein<br>K13381; bifunctional chitinase/lysozyme	[1], [4], [5]
Methane metabolism	K16154; methane monooxygenase subunit A<br>K16155; methane monooxygenase subunit B<br>K16156; methane monooxygenase subunit C<br>K16157; methane monooxygenase component A alpha chain<br>K16158; methane monooxygenase component A beta chain<br>K16159; methane monooxygenase component A gamma chain<br>K16160; methane monooxygenase regulatory protein B<br>K16161; methane monooxygenase component C<br>K16162; methane monooxygenase component D	[1], [6]

	Key enzyme	Reference
	K14028; methanol dehydrogenase (cytochrome c) subunit 1<br>K14029; methanol dehydrogenase (cytochrome c) subunit 2<br>K10944; ammonia monooxygenase subunit A<br>K10945; ammonia monooxygenase subunit B<br>K10946; ammonia monooxygenase subunit C	
**Nitrogen cycle**		
Nitrogen fixation	K02586; nitrogenase molybdenum-iron protein alpha chain NifD<br>K02588; nitrogenase iron protein NifH<br>K02591; nitrogenase molybdenum-iron protein beta chain NIfK<br>K02592; nitrogenase molybdenum-iron protein NifN<br>K02593; nitrogen fixation protein NifT<br>K02594; homocitrate synthase NifV<br>K02596; nitrogen fixation protein NifX<br>K02597; nitrogen fixation protein NifZ<br>K00531; nitrogenase AnfG	[1], [5], [7]
Assimilatory nitrate reduction	K00360; nitrate reductase (NADH) NasB<br>K00366; ferredoxin-nitrite reductase NirA<br>K00367; ferredoxin-nitrate reductase NarB<br>K00372; nitrate reductase catalytic subunit NasA	[1], [5]
Dissimilatory nitrate reduction	K00370; nitrate reductase 1, alpha subunit NarG<br>K00371; nitrate reductase 1, beta subunit NarH<br>K00373; nitrate reductase 1, delta subunit NarJ<br>K00374; nitrate reductase 1, gamma subunit NarI<br>K00362; nitrite reductase (NAD(P)H) large subunit NirB<br>K00363; nitrite reductase (NAD(P)H) small subunit NirD<br>K02567; periplasmic nitrate reductase NapA<br>K02568; cytochrome c-type protein NapB<br>K03385; cytochrome c-552 NrfA<br>K15876; cytochrome c-type protein NrfH	[1], [5]
Nitrification	K10535; hydroxylamine oxidase Hao<br>K10944; ammonia monooxygenase subunit A AmoA<br>K10945; ammonia monooxygenase subunit B AmoB<br>K10946; ammonia monooxygenase subunit C AmoC	[1], [5]
Denitrification	K15864; nitrite reductase (NO-forming) NIrS<br>K00368; nitrite reductase (NO-forming) NIrK<br>K00376; nitrous-oxide reductase NosZ<br>K02305; nitric oxide reductase subunit C NorC<br>K04561; nitric oxide reductase subunit B NorB	[1], [5]
Anammox	K10535; hydroxylamine oxidase<br>K01428; urease subunit alpha<br>K01429; urease subunit beta	[1]

	Key enzyme	Reference
	K01430; urease subunit gamma	
**Sulphate cycle**		
Assimilatory sulfate reduction	K00380; sulfite reductase (NADPH) flavoprotein alpha-component CysJ<br>K00381; sulfite reductase (NADPH) hemoprotein beta-component CysI<br>K00390; phosphoadenosine phosphosulfate reductase CysH<br>K00392; sulfite reductase (ferredoxin) Sir<br>K00860; adenylylsulfate kinase CysC<br>K00955; bifunctional enzyme CysN/CysC<br>K00958; sulfate adenylyltransferase Sat	[1], [5]
Dissimilatory sulfate reduction and oxidation	K11180; sulfite reductase, dissimilatory-type alpha subunit DsrA<br>K11181; sulfite reductase, dissimilatory-type beta subunit DsrB<br>K00394; adenylylsulfate reductase, subunit A AprA<br>K00395; adenylylsulfate reductase, subunit B AprB<br>K00958; sulfate adenylyltransferase Sat	[1], [5]
**Phosphorous cycle**		
Alkaline phosphatases	K01077; alkaline phosphatase PhoAB<br>K01113; alkaline phosphatase D PhoD	[1]
Acid phosphatases	K01078; acid phosphatase<br>K01093; 4-phytase / acid phosphatase AppA<br>K03788; acid phosphatase (class B) AphA<br>K09474; acid phosphatase (class A) phoN	[1]

Table S4. Pathogen abundance in wastewater

	DWW	UTWW
**Human pathogens**		
Acinetobacter baumannii	0.000	0.111
Acinetobacter junii	0.116	2.753
Acinetobacter johnsonii	0.089	22.958
Acinetobacter lwoffii	0.000	0.316
Bacillusthuringiensis/cereus/anthrasis	0.000	0.005
Citrobacter freundii	0.000	1.311
Clostridium perfringens	0.005	0.137
Corynebacterium ulcerans	0.000	0.032
Enterococcus faescium	0.016	0.200
Escherichia coli-Shigella dysenteriae	0.000	2.632
Klebsiella oxytoca	0.000	1.063
Moraxella canis	0.000	0.095
Moraxella osloensis	0.000	0.689
Mycobacterium cosmeticum	0.000	0.121
Mycobacterium terrae	0.000	0.005
Staphylococcus hominis	0.000	0.037
Streptococcus anginosus	0.000	0.005
Streptococcus lutetiensis	0.011	0.395
Streptococcus pneumoniae	0.005	0.047
**Plant pathogens**		
Rhizorhabdus argentea	0.000	0.000
Acidovorax valerianellae	0.000	0.032

References

[1] Kaiser, K. et al. Driving forces of soil bacterial community structure , diversity , and function in temperate grasslands and forests. Nat. Publ. Gr. 1–12 (2016). doi:10.1038/srep33696

[2] Sadhu, S. & Maiti, T. K. Cellulase Production by Bacteria : A Review. Br. Microbiol. Res. J. 3, 235–258 (2013).

[3] Ekschmitt, K., Liu, M., Vetter, S., Fox, O. & Wolters, V. Strategies used by soil biota to overcome soil organic matter stability - why is dead organic matter left over in the soil? Geoderma 128, 167–176 (2005).

[4] Beier, S. & Bertilsson, S. Bacterial chitin degradation - mechanisms and ecophysiological strategies. Front. Microbiol. 4, (2013).

[5] KEGG: Kyoto Enciclopedia for genes and genomes. http://www.kegg.jp/kegg/

[6] Prosser, J. I. in Modern Soil Microbiology (eds. van Elsas, J. D., Jansson, J. K. & Trevors, J. T.) 258–259 (CRC Press, 2007).

[7] Dai, Z. et al. Identification of Nitrogen-Fixing Genes and Gene Clusters from Metagenomic Library of Acid Mine Drainage. PLoS One 9, (2014).
